# Does the use of pre-calculated uncertainty values change the conclusions of comparative life cycle assessments? – An empirical analysis

**DOI:** 10.1371/journal.pone.0209474

**Published:** 2018-12-19

**Authors:** Yuwei Qin, Sangwon Suh

**Affiliations:** Bren School of Environmental Science and Management, University of California, Santa Barbara, California, United States of America; University of Pittsburgh, UNITED STATES

## Abstract

In life cycle assessment (LCA), performing Monte Carlo simulation (MCS) using fully dependent sampling typically involves repeated inversion of a technology matrix for a sufficiently large number of times. As the dimension of technology matrices for life cycle inventory (LCI) databases grows, MCS using fully dependent sampling is becoming a computational challenge. In our previous work, we pre-calculated the distribution functions of the entire LCI flows in the ecoinvent ver. 3.1 database to help reduce the computation time of running fully dependent sampling by individual LCA practitioners. However, it remains as a question whether the additional errors due to the use of pre-calculated uncertainty values are large enough to alter the conclusion of a comparative study, and, if so, what is the odds of such cases. In this study, we empirically tested the probability of altering the conclusion of a comparative LCA due to the use of pre-calculated uncertainty values. We sampled 10,000 random pairs of elementary flows of ecoinvent LCIs (*a*_*i*_ and *b*_*i*_) and ran MCSs (1) using pre-calculated uncertainty values and (2) using fully dependent sampling. We analyzed the distribution of the differences between *a*_*i*_ and *b*_*i*_ (i.e., *a*_*i*_−*b*_*i*_) of each run, and quantified the probability of reversing (e.g., *a*_*i*_ > *b*_*i*_ became *a*_*i*_ < *b*_*i*_) or moderating the conclusion (e.g., *a*_*i*_ > *b*_*i*_ became *a*_*i*_ ≈ *b*_*i*_). In order to better replicate the situation under a comparative LCA setting, we also sampled 10,000 random pairs of elementary flows from the processes that produce electricity, and repeated the same procedure. The results show that no LCIs derived using pre-calculated uncertainty values constitute large enough differences from those using fully dependent sampling to reverse the conclusion. However, in 5.3% of the cases, the conclusion from one approach is moderated under the other approach or *vice versa*. When elementary flow pairs are sampled only from the electricity-producing processes, the probability of moderating the conclusions increases to 10.5%, while that of reversing the conclusions remains nil. As the number of unit processes in LCI databases increases, running full MCSs in a PC-environment will continue to be a challenge, which may lead some LCA practitioners to avoid uncertainty analysis altogether. Our results indicate that pre-calculated distributions for LCIs can be used as a proxy for comparative LCA studies in the absence of adequate computational resources for full MCS. Depending on the goal and scope of the study, LCA practitioners should consider using pre-calculated distributions if the benefits of doing so outweighs the associated risks of altering the conclusion.

## Introduction

Life cycle assessment (LCA) is a tool to evaluate the environmental performance of a product [1,2]. LCA results often support corporate and public policy decisions [3–8]. When using LCA results for decision-support, it is crucial to understand the uncertainty in them [4,9,10], because lack of understanding the uncertainty behind them may materially mislead the decisions [11–13].

In general, uncertainty analysis in LCA is performed using sampling methods or analytical approaches, and the most commonly used approach is the Monte Carlo simulation (MCS) [14–23]. MCS uses random samples of input parameters following their stochastic characteristics, and runs the model repeatedly for a sufficiently large number of times to allow statistical analysis of the results [24–27]. For example, Noshadravan et al and Gregory et al performed MCS to compare two pavement designs using the distributions of expected LCA results [28,29]. These studies considered parameter uncertainty using a fully dependent sampling approach. Imbeault-Tetreault et al performed MCS for an LCA case study with nearly 900 unit processes using fully dependent sampling and compared two scenarios around the use of Global Position System (GPS) [21]. The fully dependent sampling used by Imbeault-Tetreault et al required several hours to complete the MCS. Henriksson et al conducted 1,000 Monte Carlo simulations with fully dependent sampling for a comparative LCA of Asian aquaculture products [30]. Ren et al also performed fully dependent sampling using OpenLCA, which took the team 16 hours for 1,000 Monte Carlo simulation runs on a personal computer [31]. Existing MCS packages in professional LCA software tools including SimaPro and OpenLCA can sample parameters from foreground processes and from the underlying life cycle inventory (LCI) databases [32,33].

In LCA, performing an MCS using fully dependent sampling typically involves repeated inversion of a technology matrix for each run. As the dimension of the technology matrices used in LCA databases grows, however, MCS is rapidly becoming a computational burden to lay practitioners. The ecoinvent database, which is one of the most widely used LCA databases, used to have about 5,000 processes, while the most recent version of the database, ver. 3.4 contains over 14,000 processes [34–38]. A Monte Carlo simulation using ecoinvent ver. 3.1 takes about 1 day for 1,000 runs in a personal computer environment using a Python solution for inversion based on Gaussian elimination algorithm with 16GB random access memory (RAM) and 1TB solid-state drive (SSD) [39].

The time required for each matrix inversion in a modern computer is known to have an order of *n*^2.73^ time complexity, where *n* is the dimension of a irreducible, invertible square matrix [40–42], which is generally the case in LCAs [43]. This means that doubling the dimension of a technology matrix increases the computational time at least 4.8 times. Given that the number of processes in LCI databases continues to grow, running full MCSs will increasingly become a challenge.

In 2016, the current authors published pre-calculated uncertainty values for the entire ecoinvent ver. 3.1 LCI database for the purpose of saving computation time of running a full MCS by individual users [44]. Using pre-calculated uncertainty values for LCIs, the users of LCI database do not need to invert the entire ecoinvent database, while there still is a need to invert the technology matrix for the foreground system, which is generally much smaller in dimension. In a commentary to our paper, Heijungs et al. [45] raised a concern that the use of pre-calculated uncertainty values in comparative studies ignores the dependence among background processes, leading to a large overestimation of uncertainty due to independent sampling. In our response [46], we empirically tested the argument by Heijungs et al, and found that (1) the difference in overall uncertainty characteristics in the results between fully dependent sampling and the use of pre-calculated uncertainty is small; and that (2) the use of pre-calculated uncertainty tends to underestimate, rather than overestimate, the uncertainty measured using the Geometric Standard Deviations (GSDs).

However, it remains as a question whether the additional errors due to the use of pre-calculated uncertainty values are small enough to maintain the conclusions of a comparative study, and, if not, what are the odds of misinterpreting a comparative LCA results due to the use of pre-calculated uncertainty values. In particular, the use of pre-calculated uncertainty values does ignore the presence of internal dependency within a technology matrix [19]. Henriksson and colleagues highlighted the importance of dependent sampling in understanding the distribution of comparative LCA results [47]. There are two main issues to consider. First, when performing an MCS, a data point of the same process commonly used by the two products under comparison can be perturbed independently. In principle, however, they should be perturbed in the same direction and magnitude, which is referred to as ‘dependent sampling.’ Second, in a comparative LCA setting, the distribution of the difference between the results by the two product systems being compared helps distinguish the real difference of the two results.

We agree with Hendriksson and colleagues on the theoretical superiority of fully dependent sampling, while the computational requirements for performing fully dependent sampling remains as a concern. Therefore, the objective of this paper is to empirically test the hypothesis that the use of partially independent sampling using pre-calculated uncertainty values in a life cycle inventory alters the conclusion that would have been drawn if the uncertainty values are sampled dependently.

## Materials and methods

### Two types of sampling methods

This study compared two types of sampling methods used in calculating LCIs. The first approach, partially independent sampling (PIS), used pre-calculated uncertainty characteristics that are derived using fully dependent sampling (FDS) [44]. Although these pre-calculated uncertainty characteristics such as GSDs were derived using dependent sampling, when they are applied to an comparative LCA between products A and B, each set of parameters applied to A and B are sampled independently in such a way that the same parameter that is commonly used by both A and B can be sampled at two different points within the pre-calculated distribution [44,46].

**Fig 1:**
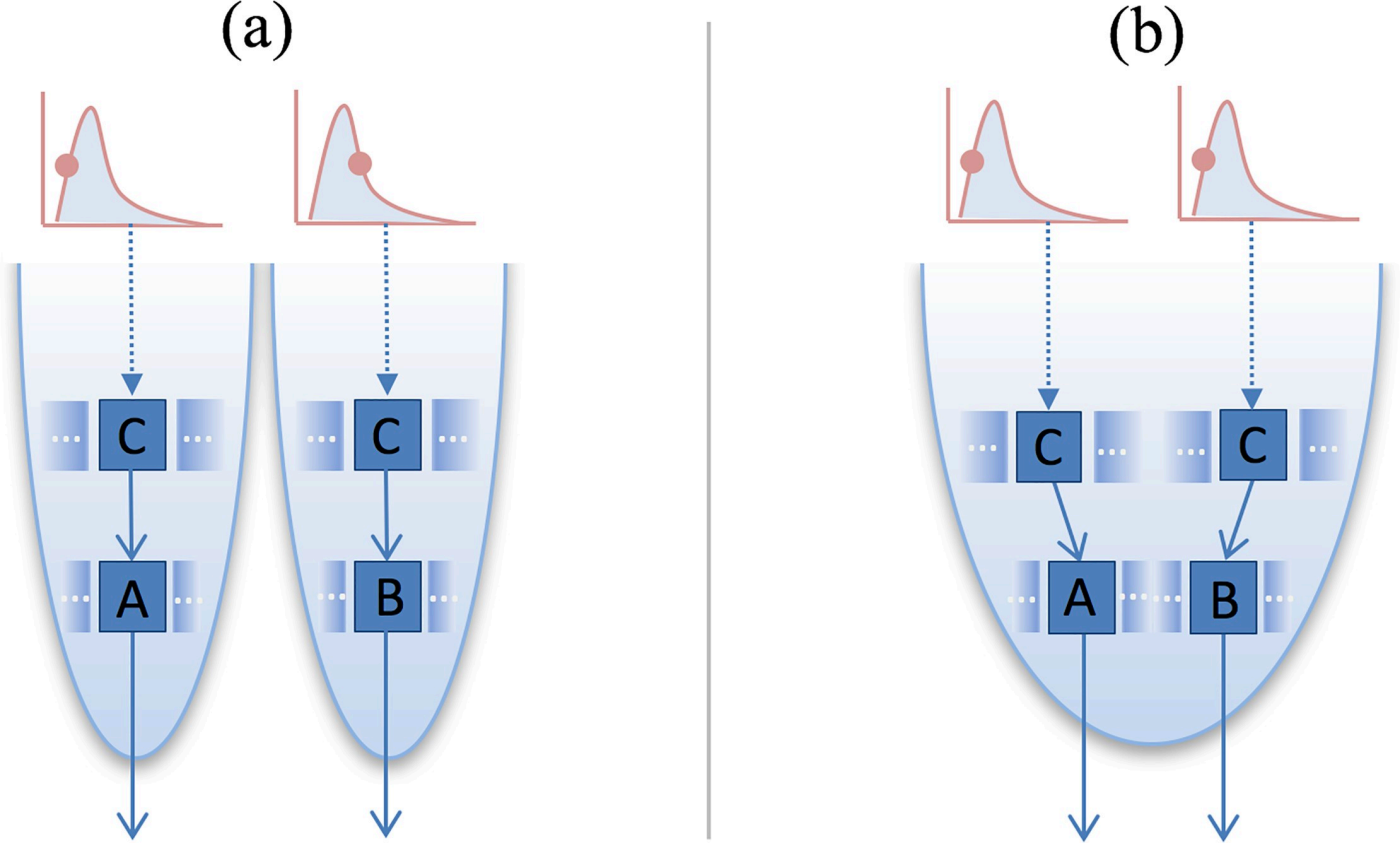
**Illustrative example of a comparative LCA between A and B involving a common input, C.** (a) partially independent sampling of the parameters involving C (use of pre-calculated uncertainty values), (b) fully dependent sampling of the parameters involving C (full Monte Carlo simulation); modified from [46]. Following the terminologies used in our previous paper, we are comparing (1) PIS (inter-input dependence with inter-product system independence; IID+IPI), which is represented in case (b) of Fig 4 in [2], with (2) FDS (inter-input dependence with inter-product system dependence; IID+IPD), which is represented in case (c) of Fig 4 in [2]. Under PIS, all parameters within each product system that produces A or B in Fig 1 are sampled dependently, while between the two product systems, a parameter commonly used by both A and B, may be sampled independently. Under FDS, all parameters of the two product systems are sampled dependently.

For example, suppose that two products produced from processes A and B are being compared. Both processes receive inputs from process C (see Fig 1(A)). When using PIS for an LCI item, e.g., CO_2_ emission for processes A and B, the randomly sampled value may be based on two different points of underlying CO_2_ emissions distribution of process C. In principle, however, the two processes should draw the same value from the distribution, if A and B are receiving the same input from the exactly same facility at the same time. Therefore, the second approach, FDS, draws the same value from process C for each run (see Fig 1(B)).

In reality, however, the parameters for process C may be derived by averaging multiple processes of different locations, and processes A and B may be using inputs from two different processes that are best represented by C in the database. In that case, the use of PIS depicted in Fig 1(A) may represent the true underlying variability in the data and can thus be justified. Conceptually, however, FDS is the ideal method used in comparing two products’ LCAs if the computation time and cost of running full Monte Carlo simulation is not considered as a barrier to LCA practitioners.

In this study, we compared the same elementary flow, *i*, in the two LCI results for A and B, which are denoted as *a*_*i*_ and *b*_*i*_, respectively. Under the FDS approach, the distribution of the difference between the two, or *a*_*i*_*—b*_*i*_, was generated using fully dependent sampling. Under the PIS approach, we used pre-calculated GSDs that were generated from FDS approach of LCIs for processes A and B. The GSDs of elementary flows were generated by sampling all processes simultaneously in the entire ecoinvent database. The use of pre-calculated uncertainty values is considered neither fully independent—because the pre-calculated values for the two products are generated from fully dependent sampling, nor fully dependently sampled—because the direct inputs and emissions of the two products are not dependently sampled. Under the PIS approach, we used the pre-calculated uncertainty values, more precisely GSDs, for sampling *a*_*i*_ and *b*_*i*_, and examined the distribution of the difference between the two.

### Distribution similarity analysis

After we ran the simulations for the comparative analysis by PIS and FDS, the distributions of LCIs from the two methods were obtained. In order to measure the similarity of the distributions of the two approaches, we used overlapping coefficient (OVL) analysis and determined the shared area between the two distributions of the difference between *a*_*i*_ and *b*_*i*_. For the given density functions *f(x)* and *g(x)*, the OVL is represented in the following equation:

OVL(f,g)=∫min{f(x),g(x)}dx

**Fig 2:**
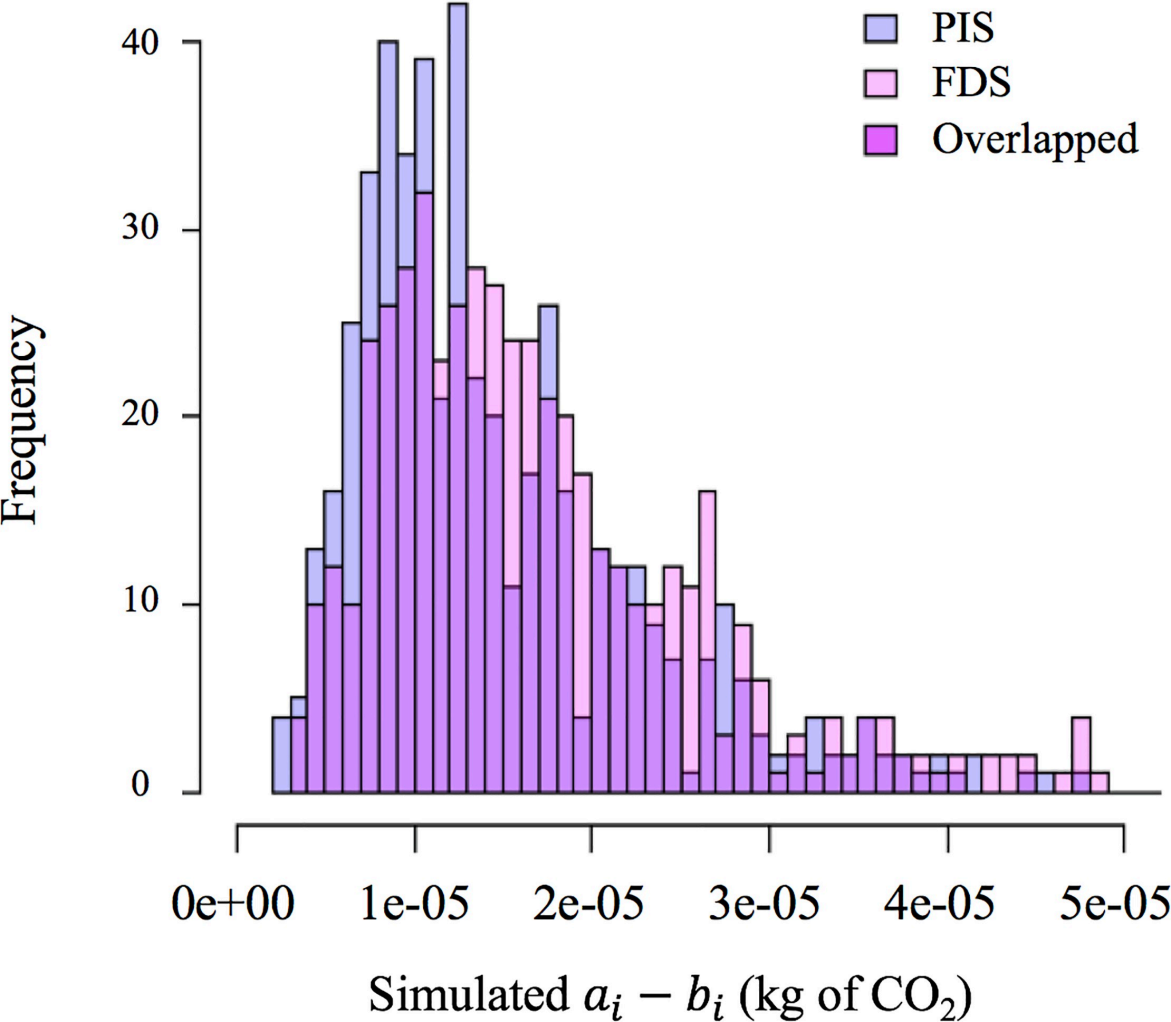
An example of overlapped histograms of one pair of elementary flows of LCIs (*a*_*i*_*—b*_*i*_*)* using PIS and FDS.

One example of overlapping coefficient is presented in Fig 2. The blue histogram represents the distribution of *a*_*i*_*—b*_*i*_ generated from the PIS approach, and the pink histogram shows the distribution of *a*_*i*_*—b*_*i*_ generated from the FDS approach. The purple area is the shared area of the two distributions, and the overlapped area can be calculated as a ratio, an overlapping coefficient. A high ratio of overlapping coefficient means the two distributions are similar to each other. The calculation of OVL for the distributions was completed in R program [48].

### Decision context

In addition to analyzing the similarity of the distributions from the two approaches, we examined the potential outcomes of comparing A and B based on the inventory item, *i* (*a*_*i*_ and *b*_*i*_). In practice, a single elementary flow is rarely, if at all, used as the basis of a comparative LCA. As we will discuss later, the use of characterized impact is likely to dampen the differences between the two sampling approaches, and therefore our use of elementary flow in this analysis should be considered as a more conservative approach; i.e., the frequency of reversing the conclusion due to the use of PIS instead of FDS would be lower if characterized results are used as the basis of a comparative LCA.

**Fig 3:**
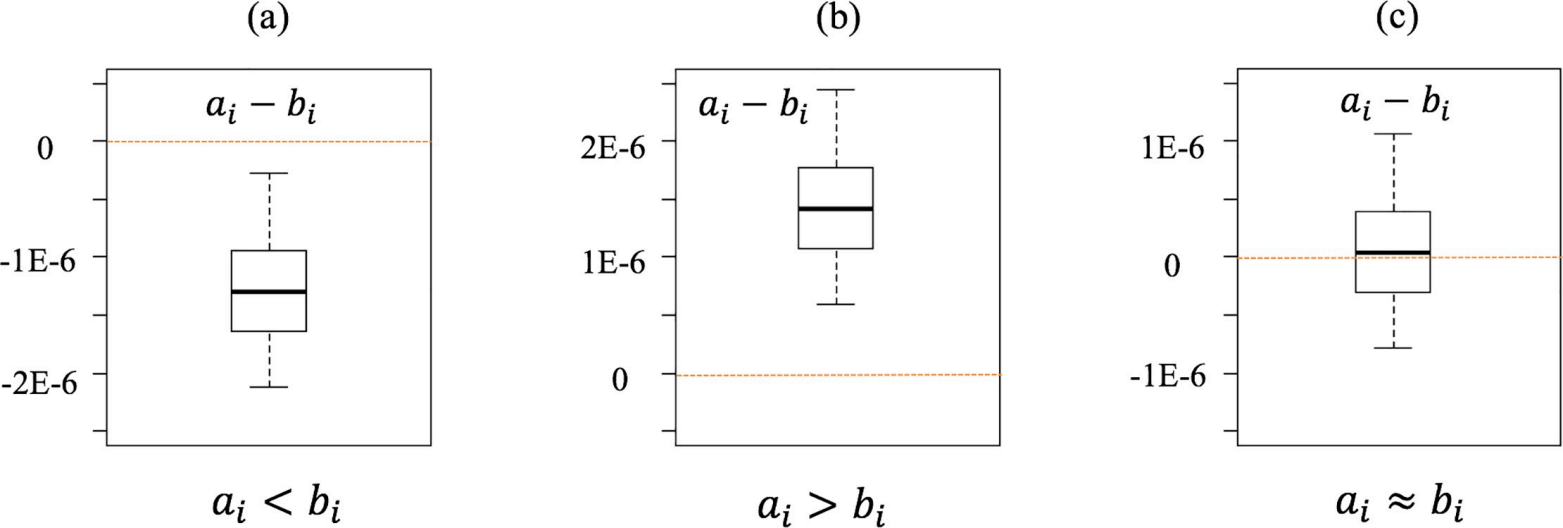
**Comparative results of LCI results for two processes A and B in the same elementary flow.** (a) A is better. (b) B is better. (c) Inconclusive conclusion.

Fig 3 shows the three possible outcomes from comparative studies. The boxplots represent the distributions of the comparative LCI results of processes A and B for the elementary flow *i* (*a*_*i*_ and *b*_*i*_).

After 1,000 random samplings and calculations for each random pair of randomly selected LCIs under MCS, we analyzed the frequency that *a*_*i*_ is smaller than *b*_*i*_. If the frequency exceeds the set threshold (70%, 80%, or 90% of the 1,000 runs), then we determined that A is better than B in terms of the elementary flow *i* (Fig 3(A)). In other words, we determine that A is better than B in terms of elementary flow *i* if *a*_*i*_−*b*_*i*_ is smaller than 0 for at least 700 runs out of 1,000 under the 70% threshold case. If the opposite is true, we determined that B is better than A (Fig 3(B)) with regard to the elementary flow. For all other cases, we determined that the comparison is inconclusive under the set threshold (Fig 3(C)).

This concept is overlaid to the use of PIS and FDS as explained in the following sections.

#### The cases that the conclusions are identical

**Fig 4:**
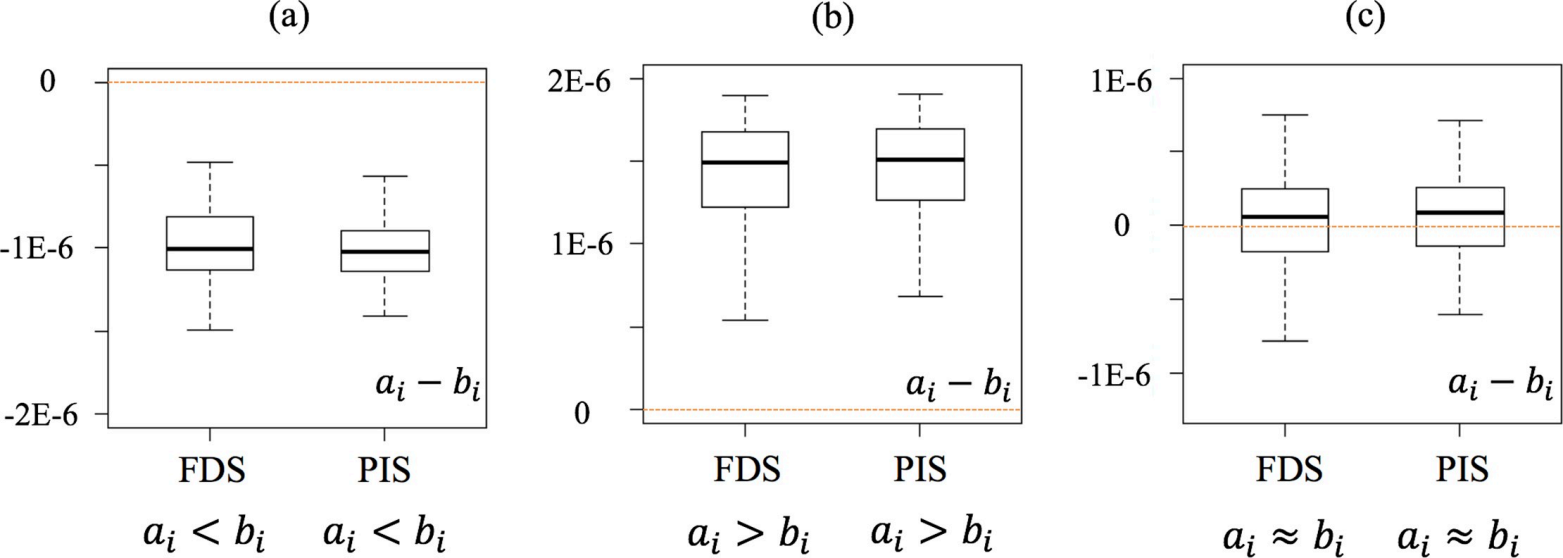
**Identical conclusions of the comparison of A and B using FDS and PIS.** (a) A is better. (b) B is better. (c) Inconclusive conclusion.

This is the case when the outcome of the comparative LCA using FDS and PIS is the same (Fig 4). In Fig 4(A), for example, the results of *a*_*i*_−*b*_*i*_ of both FDS and PIS show A is better than B within the set threshold. In this case, there is no penalty for an LCA practitioner to use the computationally lighter approach, i.e., PIS, in a comparative LCA context.

#### The cases that conclusions are moderated

**Fig 5:**
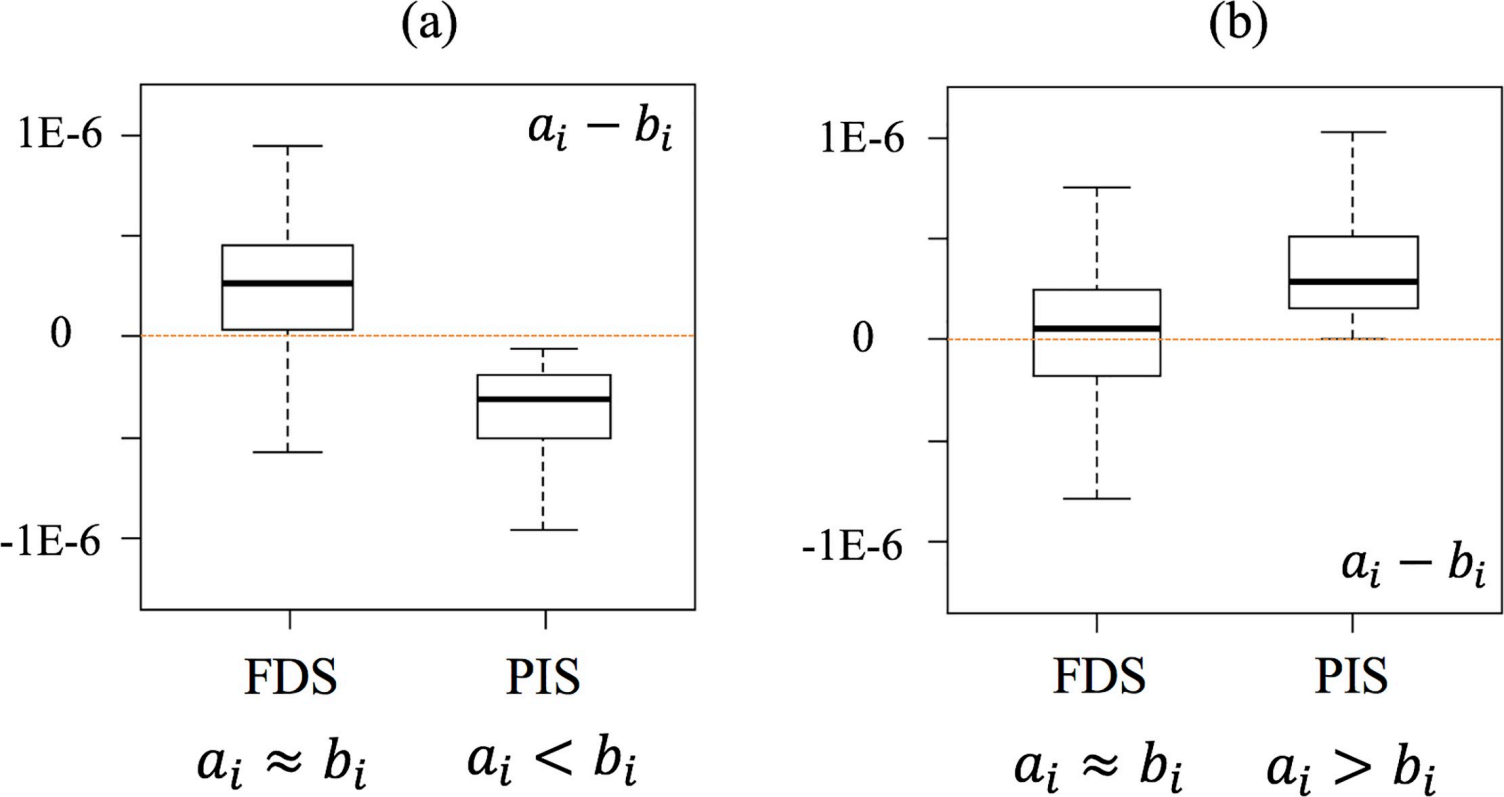
**Moderated conclusions of the comparison of A and B using FDS and PIS.** (a) One method shows A is better, and the other indicates inconclusive conclusion. (b) One method shows B is better, and the other indicates inconclusive conclusion.

This is the case when one of the two approaches (PIS or FDS) concludes that A or B is better, while the other approach concludes that the comparative outcome is inconclusive. Fig 5 shows the two cases where the conclusion is moderated by the use of PIS.

#### The cases that conclusions are reversed

**Fig 6:**
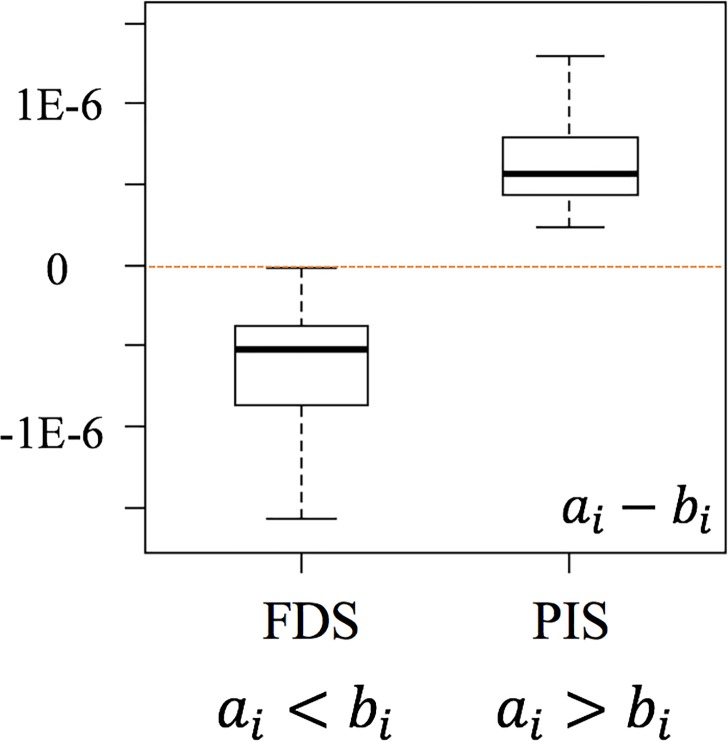
**Reversed conclusions of the comparison of A and B using FDS and PIS.** One method shows A is better, while the other indicates B is better.

The third case is that the comparative outcome obtained from FDS is reversed when PIS is used instead. For example, the results from one approach conclude that A is better than B, while the results from the other approach indicate that B is better than A within the set threshold (Fig 6).

Using the framework outlined in this section, we conducted an empirical analysis using the ecoinvent database, and the results are discussed in the next section.

## Results and discussion

### Overlapping coefficient analysis

**Fig 7:**
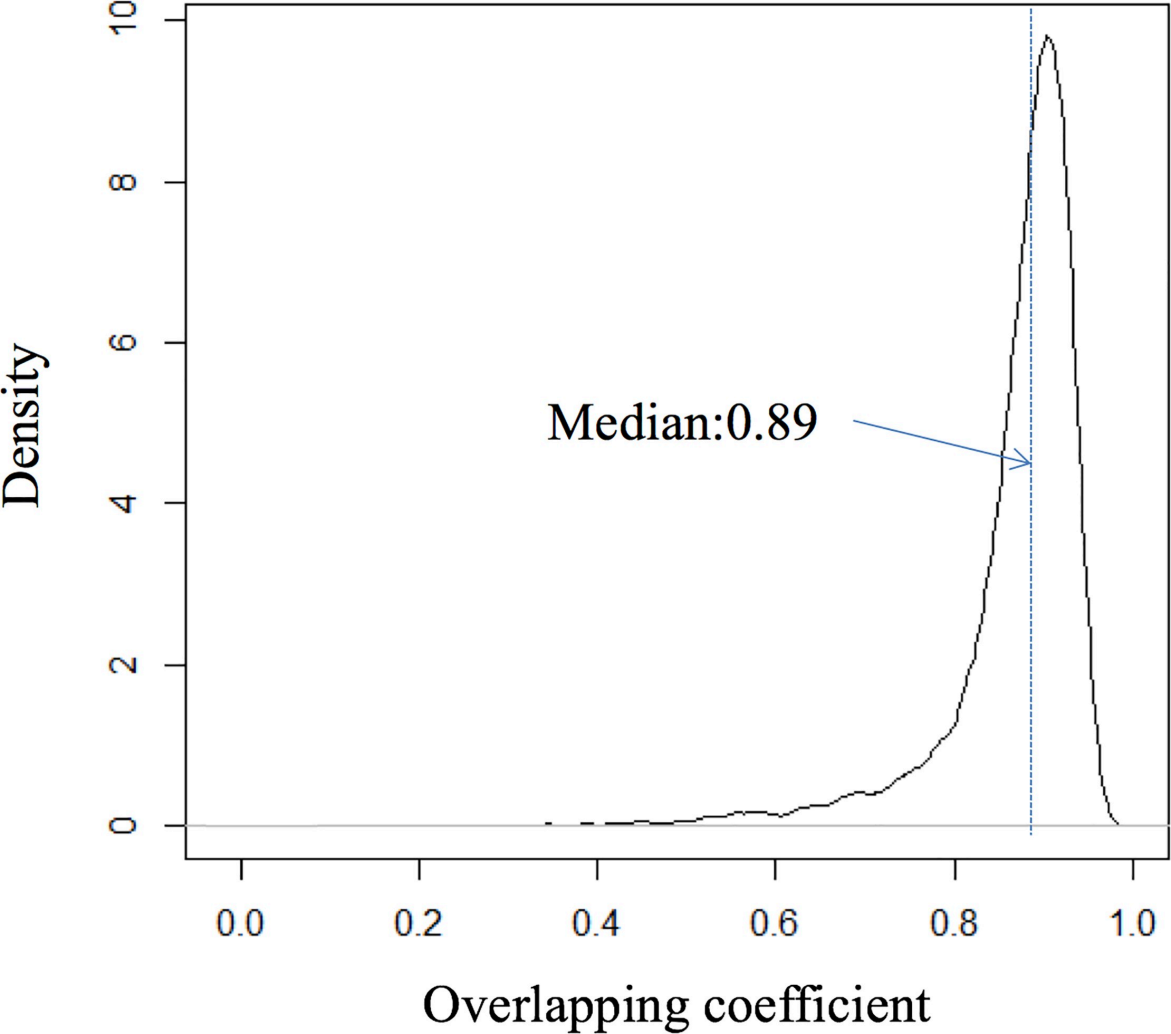
Distribution of overlapping coefficients for 10,000 pairs of elementary flows of LCIs (*a*_*i*_−*b*_*i*_) using FDS and PIS approaches.

10,000 pairs of elementary flows of LCIs, *a*_*i*_ and *b*_*i*_ were randomly selected from ecoinvent v3.1, and we simulated 1,000 times of each pair of elementary flows for both PIS and FDS approaches. Therefore, the total number of data points used for the statistical analysis was 40 million (10,000 elementary flows × 2 processes × 2 approaches × 1,000 runs). The distribution of the overlapping coefficients for 10,000 pairs of comparison is shown in Fig 7. Most overlapping coefficients (86.8%) of the distributions of FDS and PIS approaches are above 0.80, and the median is 0.89, indicating that the two methods generate similar distributions of *a*_*i*_−*b*_*i*_.

Over 74.9% of the cases showed the overlapping coefficient of 0.85 or higher indicating that the comparative results between PIS and FDS would be very similar. However, as much as 2.4% of the cases showed the overlapping coefficient of 0.6 or lower, and in those cases, the risk of drawing a different conclusion by using PIS instead of FDS is more pronounced. While OVL analysis shows the general trend of similarity between the outcomes drawn using the two approaches, the frequency of drawing a different conclusion can only be tested empirically using random sampling of actual dataset. The following section presents the result of the empirical analysis. Detailed results of the comparisons of the randomly selected processes and the processes with identical functional output are provided in S1 Table.

### Comparing randomly selected processes

**Fig 8:**
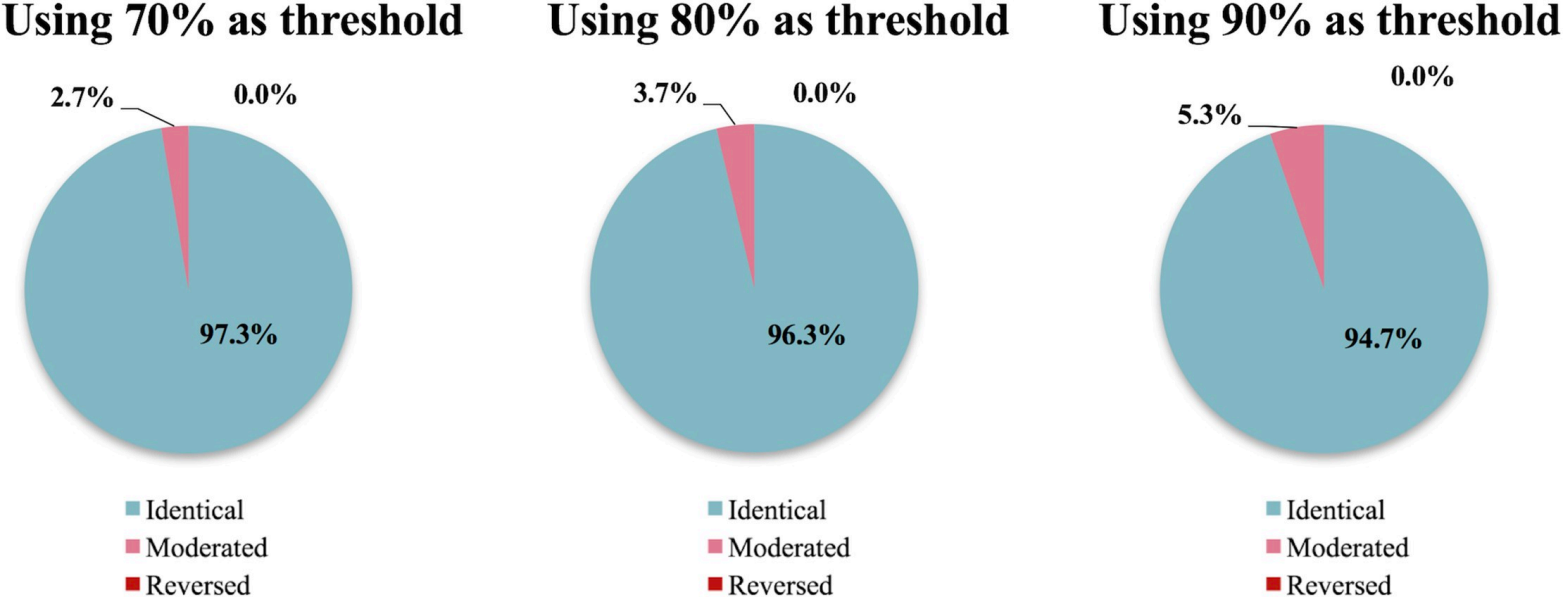
Comparison results of FDS and PIS in 10,000 pairs of random processes using 70%, 80%, and 90% thresholds.

Fig 8 shows the frequency of arriving at (1) an identical, (2) moderated, and (3) reversed conclusions by using PIS instead of FDS under three threshold conditions, 70%, 80%, and 90%. The chances that the conclusions are identical, moderated, and reversed were 94.7%, 5.3%, 0.0%, respectively when 90% was used as the threshold condition (i.e., *a*_*i*_−*b*_*i*_ should be smaller than 0 for 90% of the cases in order to determine that A is better than B). In other words, the use of pre-calculated uncertainty values generated the same results of FDS approach at about 95% of the time even when a very stringent threshold condition of 90% was employed. For the remaining 5.3%, the conclusion was moderated but not reversed.

When the threshold condition was relaxed to 80% and 70%, as expected, the chance for PIS to arrive at a moderated conclusion than the case of using FDS was reduced to 3.7% and 2.7%, respectively. Irrespective of the threshold conditions, no case out of 10,000 pairs under each threshold condition arrived at a reversed conclusion.

These results were drawn from the randomly selected processes regardless of their functional characteristics. In reality, comparative LCAs are more likely to be performed among the processes with the same or similar functional outputs. Functional equivalency of two process outputs is, however, case-dependent and often difficult to determine using only the intrinsic characteristics of the two processes. For example, polyethylene terephthalate (PET) and stainless steel are two different materials, while both of them can be used as a material for tumbler. In that sense, the results shown in Fig 8 is justifiable representation of the errors induced by PIS.

However, we also tested a more stringent case, where the processes to be compared produce the outputs of which not only the functions but also the intrinsic characteristics are equivalent. The following section follows the same procedure, while limiting the processes to be compared within electricity-producing processes, in order to see whether the same observation holds up.

### Comparing the processes with identical functional output

**Fig 9:**
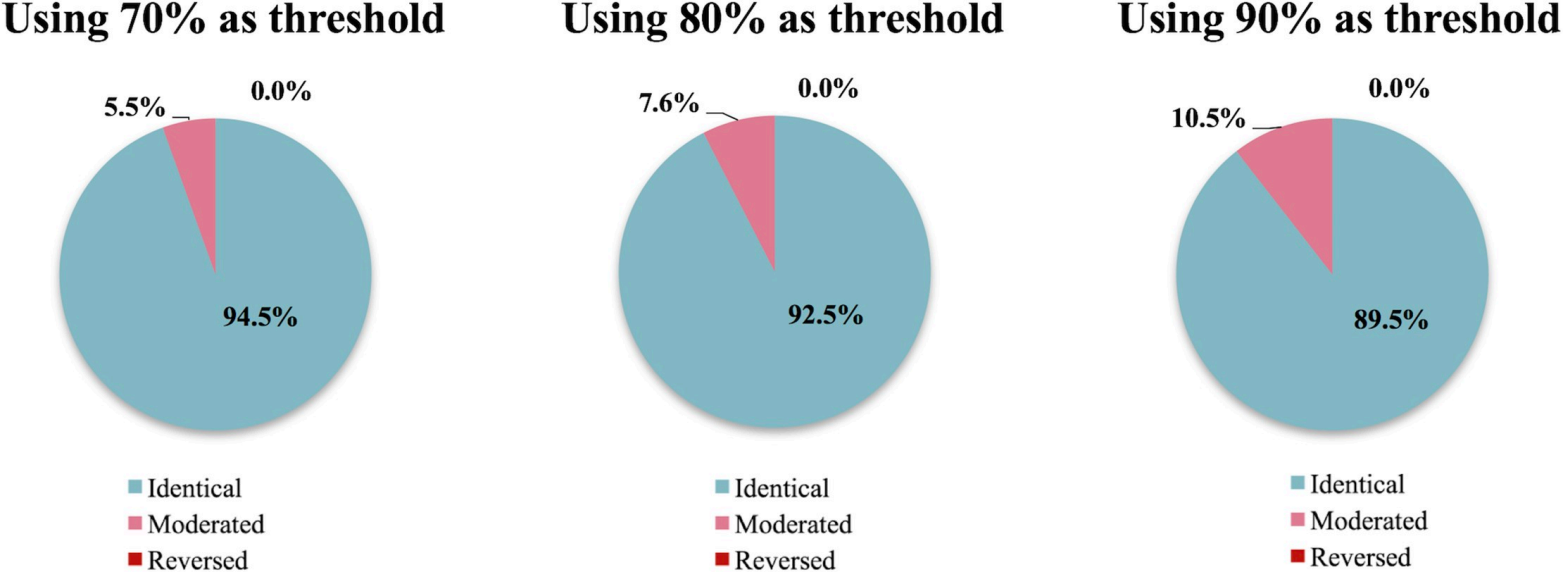
Comparison results of FDS and PIS in 10,000 pairs of random electricity processes using 70%, 80%, and 90% thresholds.

This section quantifies the frequency of arriving at a different conclusion due to the use of PIS instead of FDS among the processes that produce electricity. The results showed that the chance of arriving at a moderated conclusion by using PIS was doubled as compared to the case of randomly selected processes. However, the results still showed that most (about 90%) of the conclusions from the two methods were identical, only about 10% of the conclusions were moderated. Again, not a single case showed a reversal of the comparative outcome (Fig 9).

As was the case for the randomly sampled processes, more relaxed threshold conditions generated fewer cases where the conclusions were moderated. Table 1 shows the numerical results of the comparison between the two methods for 10,000 pairs of random sampled processes and 10,000 pairs of random sampled electricity processes. Though the number of moderated conclusions increases in the random electricity processes, the overall identical conclusions are still about 90% in the total 10,000 pairs of electricity processes.

**Table 1:** Comparison results of LCIs generated from FDS and PIS approaches in 10,000 pairs of random processes and 10,000 pairs of random electricity processes using 70%, 80%, and 90% thresholds.

	Random processes			Random electricity processes		
Threshold	70%	80%	90%	70%	80%	90%
Identical	9,733	9,629	9,467	9,451	9,245	8,947
Moderated	267	371	533	549	755	1,053
Reversed	0	0	0	0	0	0

Regardless of the similarities in the functional outcome of the processes analyzed, PIS produced the identical comparative outcome for about 9 out of 10 times when an LCI was used as the basis of the comparison with the 90% threshold condition. In the remaining 1 out of 10 cases, the results from the use of PIS have been moderated. If the processes are selected randomly or if a more relaxed threshold condition can be used, the chance for PIS to produce a moderated conclusion is reduced to 2.7% - 3.7%. If characterized or weighted results, instead of LCI, are used, the chances of moderating the conclusion by using PIS would be lower.

The question then becomes whether the benefits of using pre-calculated uncertainty values by reducing computational time and the costs associated with it outweighs the cost of added inconsistency. Certainly, this is a question that an analyst should consider given the circumstances where he or she is in, and one can hardly give a universally applicable answer to this question. For example, if an LCA practitioner is using an LCA result to claim the superiority of a produce to its competitor with a close margin, the use of PIS would not be a wise decision given the chance that it can introduce additional error in the analysis. However, for less critical cases such as LCAs for internal purposes or with limited computational power, the added errors due to the use of PIS may be acceptable. If the computational requirement for FDS is a critical barrier for performing an uncertainty analysis, certainly the benefits of using PIS would outweighs the cost of not performing uncertainty analysis.

It is also notable that the results shown in Fig 9 may not be reproducible if applied to other products with functional equivalency.

## Conclusions

Due to the growing size of LCA databases, fully dependent sampling is often a challenge to lay LCA practitioners when conducting an MCS. In this study, we evaluated the probability for an LCA practitioner to make an erroneous conclusion due to the use of pre-calculated uncertainty values or PIS instead of FDS in a comparative LCA setting. The results show that the distributions of the LCI results from the use of PIS and FDS are similar, as 86.8% of their overlapping coefficients are above 0.80. Furthermore, the chances for the use of PIS to moderate the outcomes (i.e., ‘A is better than B’ becomes ‘A and B are indifferent’, or *vice versa*) by ignoring the dependence in the upstream processes are less than 10.5% for the case of electricity-generating processes and less than 5.3% for randomly selected processes both at 90% threshold value. When the decision threshold is relaxed to 80% and 70%, the chances for the LCIs using PIS to moderate the conclusions become 3.7% and 2.7%, respectively, for randomly sampled processes and 7.6% and 5.5%, respectively, for electricity-producing processes. None of the 20,000 pairs of simulated LCIs, each of which took 1,000 runs of MCS, showed a reversal of the conclusion, which is defined in our study as the case where ‘A is better than B’ becomes ‘B is better than A,’ or *vice versa*, beyond the set thresholds (70%, 80% and 90% of the 1,000 runs).

These results are based on individual LCIs. If characterized or weighted results were used, we believe that the chances for PIS to produce erroneous conclusions may be even less pronounced that our results, given that over- and under-estimated LCIs due to the use of PIS are more likely to be cancelled out in the course of characterization and weighting.

In this paper we evaluated (1) comparisons between two randomly selected processes, and (2) comparisons between two randomly selected electricity-producing processes. The latter case presents larger number of common processes in the background between the product systems being compared, therefore the errors due to independent sampling are more pronounced. Even more extreme case would be to compare two slight design changes for the same product. In that case, there will be much more significant overlap in the upstream processes. However, those overlaps would already occur at the direct inputs to the foreground process under study, in which case they can always excluded from the comparison, as those common inputs do not contribute to the difference between the two designs. By excluding them, an LCA practitioners are essentially practicing fully dependent sampling for those common inputs. Therefore, the dependence that this paper is concerned is that within the upstream processes modelled within LCA databases, not that in direct inputs to a foreground process, which is better be simply excluded from a comparison.

Our results indicate that pre-calculated uncertainty values can be used as a proxy for understanding the uncertainty and variability in a comparative LCA study especially when adequate computational resources are lacking. The number of unit processes is increasing for many LCI databases, adding to the challenge of running MCSs in a PC-environment in the future. LCA practitioners will need to evaluate whether the additional chances of altering the conclusion due to the use of pre-calculated uncertainty values is tolerable given the goal and scope of the study. The additional errors due to the use of pre-calculated uncertainty values shown in our study seem justifiable if the alternative is no uncertainty analysis due to the lack of computational resources needed for fully dependent sampling.

We believe that the concept of using pre-calculated distributions might be applicable to other related fields such as input-output analysis and material flow analysis, potentially saving computation time and costs.

## Supporting information

S1 Table Results of 10,000 random pairs of elementary flows and 10,000 random pairs of elementary flows within the processes that produce electricity from ecoinvent. (XLSX)
